# Insights Into Metabolic Signatures and Regulatory Effect of *Dendrobium officinale* Polysaccharides in Gut Microbiota: A Comparative Study of Healthy and Diabetic Status

**DOI:** 10.1002/fsn3.4651

**Published:** 2024-12-06

**Authors:** Qianbo Song, Junju Zou, Sau Wan Cheng, Kendra Sek Lam Li, David Tai Wai Lau, Xiao Yang, Pang Chui Shaw, Zhong Zuo

**Affiliations:** ^1^ Guangdong‐Hong Kong‐Macao Joint Laboratory for New Drug Screening, School of Pharmacy The Chinese University of Hong Kong Hong Kong SAR P. R. China; ^2^ State Key Laboratory of Research on Bioactivities and Clinical Applications of Medicinal Plants The Chinese University of Hong Kong Hong Kong SAR P. R. China; ^3^ School of Life Sciences and Li Dak Sum & Yip Yio Chin Research and Development Centre for Chinese Medicine The Chinese University of Hong Kong Hong Kong SAR P. R. China; ^4^ School of Traditional Chinese Medicine Hunan University of Chinese Medicine Chang Sha P. R. China; ^5^ Department of Microbiology The Chinese University of Hong Kong Hong Kong SAR P. R. China

**Keywords:** *Dendrobium officinale* polysaccharides, diabetes, different status, fermentation, gut microbiota, metabolic

## Abstract

*Dendrobium officinale* is a kind of popular functional food to be consumed by both healthy and diabetic people. As its major constituent, *D. officinale* polysaccharide (DOP) is mainly utilized by gut microbiota. Despite distinctive gut microbiota composition between healthy and diabetic individuals, no study compared the interplay between DOP and gut microbiota under healthy and diabetic status. The current study aims to investigate and compare the metabolic signatures and regulatory potential of DOP on gut microbiota between healthy and diabetic status. Our serial in vitro fermentation investigations found that mannose in DOP was more utilized by gut microbiota under diabetic status with higher production of propanoic acid and lower production of butyric acid compared with those under healthy status. Moreover, metabolomic analyses revealed different impacts of DOP on intestinal microbial metabolites between healthy and diabetic status with upregulating taurine and downregulating 2‐hydroxybutyric acid only occurring under diabetic status. Biodiversity analyses demonstrated that DOP treatment could only significantly improve the diversity of gut microbiota under diabetic status while there was no significant effect on that under healthy status. Further gut microbiota composition analyses indicated that DOP treatment could promote probiotics (*Dubosiella*, *Bifidobacterium*, and *Akkermansia*) under both healthy and diabetic status while inhibit pathogens (*Escherichia‐Shigella*) only under diabetic status. In summary, our current insights into metabolic signatures and regulatory effects of DOP in the gut microbiota under healthy and diabetic status provided scientific evidence for its broad use as functional food.

## Graphical abstract

Different metabolic fate and regulatory effect of DOP in gut microbiota under healthy and diabetic status.

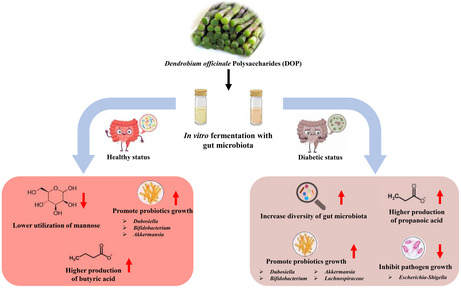

**Abbreviations**
DEN: *Dendrobium officinale*
DOP: *Dendrobium officinale* polysaccharides
GC–MS: gas chromatography–mass spectrometry
GMD: gut microbiota under diabetic status
GMH: gut microbiota under healthy status
SCFA: short‐chain fatty acids
T2D: type‐II diabetes
VIP: variable importance in the projection

## 1 Introduction

As a functional food, the stem of *Dendrobium officinale* (DEN) is commonly consumed as beverage by both chronic disease patients and healthy individuals in Asia for decades owning to its diverse health benefits. It could not only improve intestinal microenvironment of healthy individuals by promoting their probiotic growth (Sun et al. 2023) but also exhibit therapeutic potentials on patients with cancer, colitis, and metabolic disorders (Chen, Nie, et al. 2021; Chen, Wu, et al. 2021). Type‐II diabetes (T2D) is one typical metabolic disease accompanied with disordered blood glucose metabolism. According to the recommendation from China Nutrition and Health Food Association, DEN could be used in the adjuvant therapy of T2D and its complications. A market survey in China also indicated that DEN was commonly made into tea to be consumed by T2D patients for alleviating their disordered glucose metabolism (Li et al. 2017).

As one indispensable part of intestinal microenvironment, gut microbiota is deeply associated with many physiological processes in the host, and its dysbiosis may result in the dysfunction of human body and occurrence of disease. Recently, it was found that polysaccharides were important constituents in natural functional foods and often exhibited therapeutic potential on metabolic disordered diseases like T2D by regulating gut microbiota in the host (Yang, Chen, et al. 2020; Yang, Yang, et al. 2020). In fact, most edible polysaccharides such as Tibetan tea polysaccharides, straw mushroom polysaccharides, and kiwi fruit polysaccharides passed through the digestive system intactly without degradation by gastrointestinal digestive enzymes and encountered gut microbiota in the large intestine, where polysaccharides would be utilized by gut microbiota and conversely regulate gut microbiota composition as well (Chen, Chen, et al. 2023; Chen, Liu, et al. 2023; Hu et al. 2023; Tan et al. 2023; Guo et al. 2022).

*Dendrobium officinale* polysaccharides (DOP), as the main bioactive constituents of DEN, have obtained increasing interest in recent decades because of its health‐promoting effects including anti‐inflammation, anti‐hyperlipidaemia and anti‐osteoporosis (Wang, Jiang, et al. 2023; Wang, Wang, et al. 2023; Wang, Zhang, et al. 2023; Zhou et al. 2023; Yang, Fu, et al. 2024; Yang, Li, et al. 2024). Glucomannan, one of the major constituents of DOP, was found to be able to regulate the glucose and lipid metabolism in T2D rats via promoting the growth of *Parabacteroides*, *Bifidobacterium*, and *Faecalibacterium* (Chen, Nie, et al. 2021; Chen, Wu, et al. 2021). In addition, a homogeneous polysaccharide recently isolated from DEN with the molecular weight of 2.3 × 10^5^ Da also exhibited beneficial effects on T2D mice by enhancing the abundance of *Bifidobacterium* and *Lachnospiraceae* (Li et al. 2024). As another study (Li et al. 2019) and our preliminary investigation consistently indicated that DOP remained intact after gastric and small intestinal digestion as shown in Figure S1, it was highly suggested that large intestine served as an important site for DOP, and gut microbiota in the large intestine might be involved in its therapeutic effect.

Since clinical evidence indicated different profiles of gut microbiota composition between T2D and healthy individuals (Wang et al. 2012), it was hypothesized that there might be different interplays between DOP and gut microbiota under healthy status (GMH) and diabetic status (GMD). To our best knowledge, studies comparing the metabolic signatures of DOP and its regulation potential on gut microbiota between healthy and diabetic status were absent. Therefore, the present study adopted an in vitro fermentation system not only for the first time to reveal different metabolic signatures of DOP after fermentation with GMH and GMD but also evaluate its regulatory impact on gut microbiota under healthy and diabetic status.

## 2 Materials and Methods

### 2.1 Materials and Reagents

Fresh *D. officinale* was purchased from the local market of Hong Kong and authenticated by Dr. Lau Tai Wai from School of Life Science, The Chinese University of Hong Kong. Glucose, mannose, 1‐phenyl‐3‐methyl‐5‐pyrazolone, and trimethyl acetic acid were purchased from Chroma Biotech Co. Ltd. (Chengdu, China). Dextran standards with different molecular weights (MW: 1200–2,556,000 Da) were obtained from American Polymer Standards (Mentor, USA). Standards of three short‐chain fatty acids (acetic acid, propanoic acid, and butyric acid) were from Sigma‐Aldrich (Saint Louis, USA). The purity of all the reagents were more than 98%, and all the organic solvents were in HPLC grade. Distilled water was prepared by a Millipore water purification system (Milford, MA, USA).

### 2.2 Preparation and Chemical Characterizations of DOP

The stem of fresh DEN was cut into pieces and baked in the oven at 40°C. The obtained dry DEN stem was boiled with distilled water for 1 h at the ratio of 0.1 kg/L, and the extraction was repeated twice. Then, the extract was combined and centrifuged (4500 rpm, 15 min), and the supernatant was lyophilized to obtain DOP. As shown in Figure S2, our prepared sample was mainly composed of polysaccharides with the total sugar content of 90.8% ± 2.7%. The result of monosaccharide composition analysis shown in Figure S3 indicated that our prepared DOP was composed of mannose and glucose at the molar ratio of 2.12:1.00. Molecular weight distribution analysis on our prepared DOP shown in Figure S4 indicated its molecular weight ranged from 300 to 6 × 10^5^ Da.

### 2.3 In Vitro Fermentation of DOP With GMH and GMD

#### 2.3.1 Preparation of Fecal Slurry Fluid

Fresh fecal samples from *db*/*db* mice (6‐week‐old, *n* = 6) and C57BL/6J mice (6‐week‐old, *n* = 6) free from any medicine treatment were pooled and kept at sterile tubes. The collection of fecal samples was approved by Animal Experimentation Ethics Committee of The Chinese University of Hong Kong (No.:21/167/LDS). The collected feces (4 g) were homogenized with 0.1 mol/L sterile PBS buffer (40 mL), followed by centrifugation (3500 rpm, 10 min, 4°C) to collect the supernatant for subsequent experiments.

#### 2.3.2 In Vitro Fermentation of DOP With Prepared Fecal Slurry Fluid

The in vitro fermentation of DOP with GMH or GMD was conducted based on previously reported procedures with modification (Chen et al. 2018; Dou, Chen, and Fu 2019). First, general anaerobic media with DOP was prepared by dissolving 120 mg DOP into 21.6 mL blank general anaerobic media. Then, 2.4 mL of the prepared fecal slurry fluid from *db*/*db* mice was added into autoclaved general anaerobic media (21.6 mL) with and without DOP, and they were labeled as the DOP‐GMD group and Blank media‐GMD group, respectively. Similarly, autoclaved general anaerobic media (21.6 mL) with and without DOP were mixed with 2.4 mL of the prepared fecal slurry fluid from C57BL/6J mice and labeled as the DOP‐GMH group and Blank media‐GMH group, respectively. Blank general anaerobic media and inulin worked as blank control and positive control, respectively. The recipe of general anaerobic media is listed in Table S1. All the broth were anaerobically incubated at 37°C for 48 h, and fermentation was conducted in triplicate. The sampling and pH measurement were performed at 0, 8, 24, and 48 h. All the samples were stored at −80°C before analysis.

### 2.4 Comparison on Metabolic Signatures of DOP After Fermentation With GMH and GMD

#### 2.4.1 Chemical Characterizations of DOP Before and After Fermentation

##### 2.4.1.1 Measurement of Total Sugar Content

The total sugar content of DOP or the supernatant from its fermentation broth was quantified by anthrone‐sulfuric acid method with glucose as reference. The extent of DOP utilized by gut microbiota was indicated by residual sugar ratio which was calculated by the following formula:

$$\text{Residual sugar ratio}=T_{n}/T_{0}$$

where *T*_0_ and *T*_*n*_ were the total sugar content at initial and subsequent timepoints, respectively.

##### 2.4.1.2 Molecular Weight Distribution Analysis

DOP or the supernatant of its fermentation broth was filtered by a 0.45 μm filter, followed by injecting the filtrate into a U‐3000 HPLC system coupled with a charged aerosol detector (Thermofisher, MA, USA). The alteration of molecular weight distribution was profiled on a TSK GMPWXL gel permeation column (7.8 × 300 mm; Tosoh Bioscience) with a mobile phase of 20 mM ammonium acetate at the flow rate of 0.6 mL/min.

##### 2.4.1.3 Monosaccharide Composition Analysis

The monosaccharide composition of DOP during the fermentation process was monitored by the modified 1‐phenyl‐3‐methyl‐5‐pyrazolone derivative method (Kuang et al. 2020). In brief, the fermentation broth of DOP (0.5 mL) was hydrolyzed with trifluoroacetic acid, followed by evaporation to remove acid. About 100 μL of the obtained hydrolysate solution or standard monosaccharide solution (1 mg/mL) was alkalized with 100 μL NaOH solution (0.6 mol/L) and reacted with 200 μL 1‐phenyl‐3‐methyl‐5‐pyrazolone solution (0.5 mol/L) at 70°C for 2 h. The resultant solution was diluted with 1 mL distilled water and neutralized with 100 μL HCl solution (0.6 mol/L), followed by extraction with chloroform (1.5 mL). The remaining aqueous phase was filtered through a 0.22 μm filter and analyzed by an Agilent 1260 HPLC system (Santa Clara, USA) coupled with an Eclipse‐XDB‐C_18_ column (5 μm, 250 × 4.6 mm, Agilent) based on the chromatographic condition described previously (Song et al. 2022).

#### 2.4.2 Comparison on Metabolic Profiles of DOP Fermented With GMH and GMD

##### 2.4.2.1 Untargeted Metabolomic Analysis by UPLC‐Orbitrap Mass Spectrometry

The supernatant (100 μL) of DOP fermentation broth was mixed with 400 μL of organic solvent (methanol: acetonitrile = 1:1) and sonicated for 10 min in an ice‐water bath, followed by standing still (4°C for 1 h) to precipitate proteins. Then, the mixture was centrifuged (12,000 rpm, 15 min, 4°C), and the supernatant was taken out, followed by mixing with an internal standard and injecting into a UPLC‐Orbitrap Exploris 120 mass spectrometer system (Thermofisher Scientific) coupled with a Waters ACQUITY UPLC HSS T3 C_18_ column (2.1 mm × 100 mm, 1.8 μm). The mobile phase consisted of (A) aqueous solution (containing 5 mmol/L ammonium acetate buffer) and (B) acetonitrile at the flow rate of 0.35 mL/min, with the gradient program listed in Table S3. The mass spectrometry data were acquired based on information‐dependent acquisition mode, and its analytical parameters were set as follows: Sheath gas flow rate as 50 Arb, Aux gas flow rate as 15 Arb, and capillary temperature of 320°C. The resolution of full MS and secondary MS was set as 60,000 and 15,000, respectively. The sample was analyzed under positive and negative ionization modes with the spray voltage of 3.8 and −3.4 kV, respectively. The raw mass spectra were converted by ProteoWizard software for peak detection, extraction, alignment, and integration. The metabolites were identified by matching the high‐resolution MS and MS/MS fragment information with the in‐house database.

##### 2.4.2.2 Short‐Chain Fatty Acid Analysis by Gas Chromatography–Mass Spectrometry

Gas chromatography–mass spectrometry (GC–MS) was used to quantify the level of short‐chain fatty acids (SCFA) in the DOP fermentation broth according to the reported method (Tsang et al. 2018). After centrifuging the fermented samples (3500 rpm, 10 min, 4°C), the obtained supernatant (720 μL) was pipetted out and deionized with 1% formic acid (80 μL), followed by extracting with ethyl acetate. Then, the organic solvent phase was filtered by a 0.22 μm filter and mixed with trimethyl acetic acid (internal standard, *v*:*v*, 1:1) and injected into a Shimadzu QP2010 GC–MS system. The standard stock solution was serially diluted and made to undergo the same pretreatment for plotting the standard curve. The GC–MS analytical parameters are listed in Table S4.

### 2.5 Comparison on the Composition Profiles of GMH and GMD After DOP Fermentation

The broth of DOP that underwent 48 h fermentation with GMH and GMD was collected, followed by centrifugation (4500 rpm, 15 min) to obtain the precipitates. The total DNA of microbes in the broth was extracted from the precipitates by the CTAB method, and DNA integrity was determined by agarose gel electrophoresis. The sequence information of gut microbiota was identified by the 16S ribosomal RNA method. The V3–V4 domain of the bacterial gene was selected and amplified with the primer of 341F (5′‐CCTACGGGNGGCWGCAG‐3′) and 805R (5′‐GACTACHVGGGTATCTAATCC‐3′). The obtained PCR products were purified by the AMPure XT beads kit (Beckman Coulter Genomics, Danvers, MA, USA). Their sequence information was generated by the HiSeq 2500 PE250 platform. The obtained sequence raw data were denoised with the DADA2 method and identified with the database of SILVA and NT‐16S.

### 2.6 Data Analyses

GraphPad Prism 9.0 software (GraphPad, USA) was used for statistical analyses. All the experiment values were expressed as mean ± SD. Comparisons on statistical difference between groups were conducted by one‐way analysis of variance with Turkey's test. The metabolomic data were analyzed by R package with Pareto‐scaled principal component analysis and orthogonal partial least‐squares discriminant analysis. The sevenfold cross‐validation and response permutation test was adopted to evaluate the robustness of the model. The variable importance in the projection (VIP) value of each variable in the orthogonal partial least‐squares discriminant analysis model was calculated to indicate its contribution to the classification. Student's t test was applied to determine the significance of differences between groups, and the metabolites (VIP > 1 and *p* < 0.05) were determined with significant difference. Spearman's correlation analysis between the relative abundance of gut microbiota and the level of nonvolatile metabolites as well as SCFA was performed among Blank media‐GMH, DOP‐GMH, Blank media‐GMD, and DOP‐GMD groups.

## 3 Results and Discussion

DOP was verified to be the main bioactive constituent of DEN stem as shown in Figure S2, and its metabolic signatures and regulatory effect in GMH and GMD were investigated and compared for the first time in the current study. The *db*/*db* mice are the commonly used diabetic mice with the mutation of the leptin receptor gene located at chromosome 4, and they normally spontaneously exhibit hyperglycemia with the disordered gut microbiota community since the age of 5–6 weeks (Xavier and Hodson 2018). In the current study, GMH and GMD were isolated from wild‐type C57BL/6J mice and *db*/*db* mice, respectively, and applied in the in vitro fermentation model to monitor and compare their interplay with DOP.

### 3.1 Different Chemical Alterations of DOP After Its Fermentation With GMH and GMD

**FIGURE 1:**
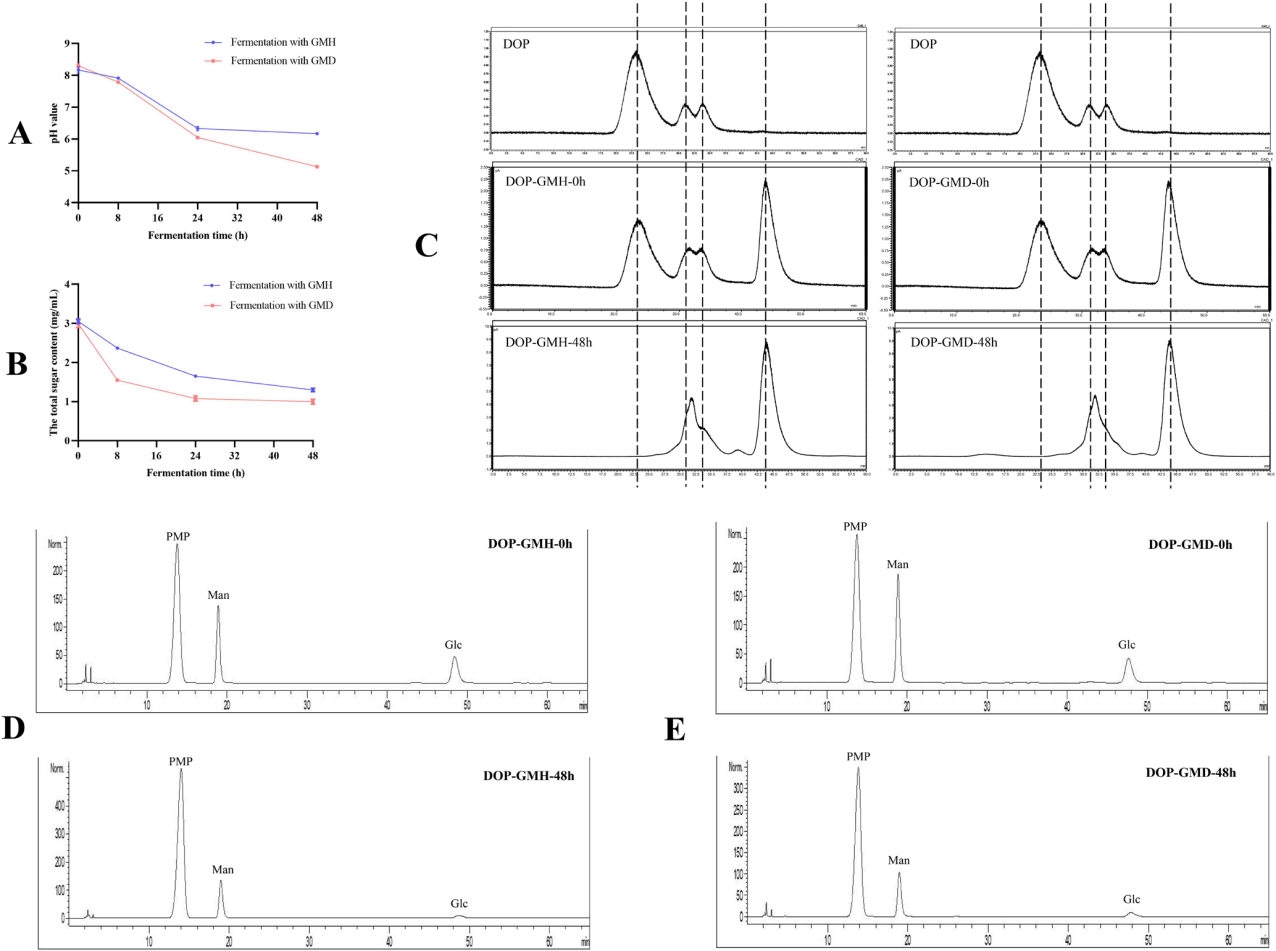
Alteration of pH value (A), total sugar level (B), molecular weight distribution profile (C), and monosaccharide composition of DOP broth (D, E) before and after fermentation by GMH and GMD.

As shown in Figure 1A, the pH value of DOP broth fermented with both GMH and GMD decreased with the extension of fermentation time. In addition, it was found that the total sugar content of DOP was also decreased with the ratio of 42.54% ± 1.22% and 33.43% ± 3.09% after 48 h fermentation by GMH and GMD, respectively (Figure 1B). In comparison with the fermentation with GMH, the DOP broth exhibited lower total sugar content and pH value after 48 h fermentation with GMD, suggesting that more DOP was utilized by GMD than that by GMH. Molecular weight distribution analysis (Figure 1C) indicated that the molecular weight of DOP was distributed at 6.0 × 10^5^, 4000, and 1000 Da prior to fermentation. After 48 h fermentation, the molecular weight profile of DOP fermented with GMD and GMH were quite similar with only two peaks remaining at around 1500 and 200 Da. The absence of macromolecular peak (around 6.0 × 10^5^ Da) of DOP might be degraded by gut microbiota.

Further monosaccharide composition analysis shown in Figure 1D and Table 1 indicates that DOP was composed of mannose and glucose at the molar ratio of 2.10:1.00. After 48 h fermentation with GMH and GMD, the mannose level in DOP decreased to 4.037 ± 0.260 μmol/mL and 3.338 ± 0.078 μmol/mL, respectively, while the glucose level in DOP decreased to 0.747 ± 0.063 μmol/mL and 0.708 ± 0.040 μmol/mL, respectively. It is suggested that both mannose and glucose in DOP could be utilized by gut microbiota regardless of the pathological status of the host. In addition, the molar ratio of mannose to glucose in DOP was found to be significantly increased to 5.408 ± 0.120 and 4.728 ± 0.328 after 48 h fermentation with GMH and GMD, respectively. The increased molar ratio of mannose to glucose after fermentation suggested that glucose in DOP was much easier to be utilized by gut microbiota than mannose regardless of the pathological status of the host, which was supported by a previous finding that gut microbiota exhibited preference on the utilization of saccharides, and it was dependent on the types of hydrolysis enzymes in gut microbiota and the structural properties of polysaccharides such as molecular weight, glycosidic linkage, and monosaccharide composition (Li et al. 2023). Furthermore, the utilization manner on monosaccharides in DOP was also compared between GMD and GMH; it was found that mannose in DOP was more significantly utilized by GMD than that by GMH, whereas no significant difference was observed on that of glucose, suggesting that gut microbiota under different pathological status might exhibit differential utilization manner on the monosaccharides in DOP. Such phenomenon was supported by a similar finding that gut microbiota from host under different physiological status exhibited differential utilization manner on inulin (Chen, Chen, et al. 2023; Chen, Liu, et al. 2023). To our best knowledge, most studies investigated the metabolic signature of edible polysaccharides in the simulated fermentation model with gut microbiota from the healthy host (Bisht, Goh, and Merino‐Matia 2023). Few studies compared the metabolic signature of edible polysaccharides after interplaying with gut microbiota from healthy or diabetic host. The present study for the first time demonstrated that the metabolic signatures of DOP were different after fermentation with GMH and GMD, providing an insight that host pathological status might impact the interplay between gut microbiota and polysaccharides from functional food like DOP.

**TABLE 1:** Comparison on monosaccharide composition of DOP fermented with gut microbiota under healthy and diabetic status.

Status	Timepoints (h)	Monosaccharide composition of DOP before and after fermentation				
		Mannose (μmol/mL)	Utilization ratio of mannose	Glucose (μmol/mL)	Utilization ratio of glucose	The molar ratio of mannose/glucose
Healthy	0	5.925 ± 0.245	—	2.917 ± 0.125	—	2.035 ± 0.155
	48	4.037 ± 0.260***	31.9% ± 2.2%	0.747 ± 0.063***	74.3% ± 2.8%	5.408 ± 0.120***
Diabetic	0	5.843 ± 0.156	—	2.742 ± 0.073	—	2.131 ± 0.019
	48	3.338 ± 0.078***^,#^	42.8% ± 2.5%^##^	0.708 ± 0.040***	74.2% ± 0.9%	4.728 ± 0.328***^,#^

***p < 0.001, compared with the corresponding initial concentration; ^#^ *p* < 0.05; ^##^ *p* < 0.01, compared with fermentation under healthy status.

### 3.2 Different Regulatory Impacts of DOP on Microbial Metabolites Produced by GMH and GMD

#### 3.2.1 Different Regulatory Impacts of DOP on Nonvolatile Metabolites Produced by GMH and GMD

**FIGURE 2:**
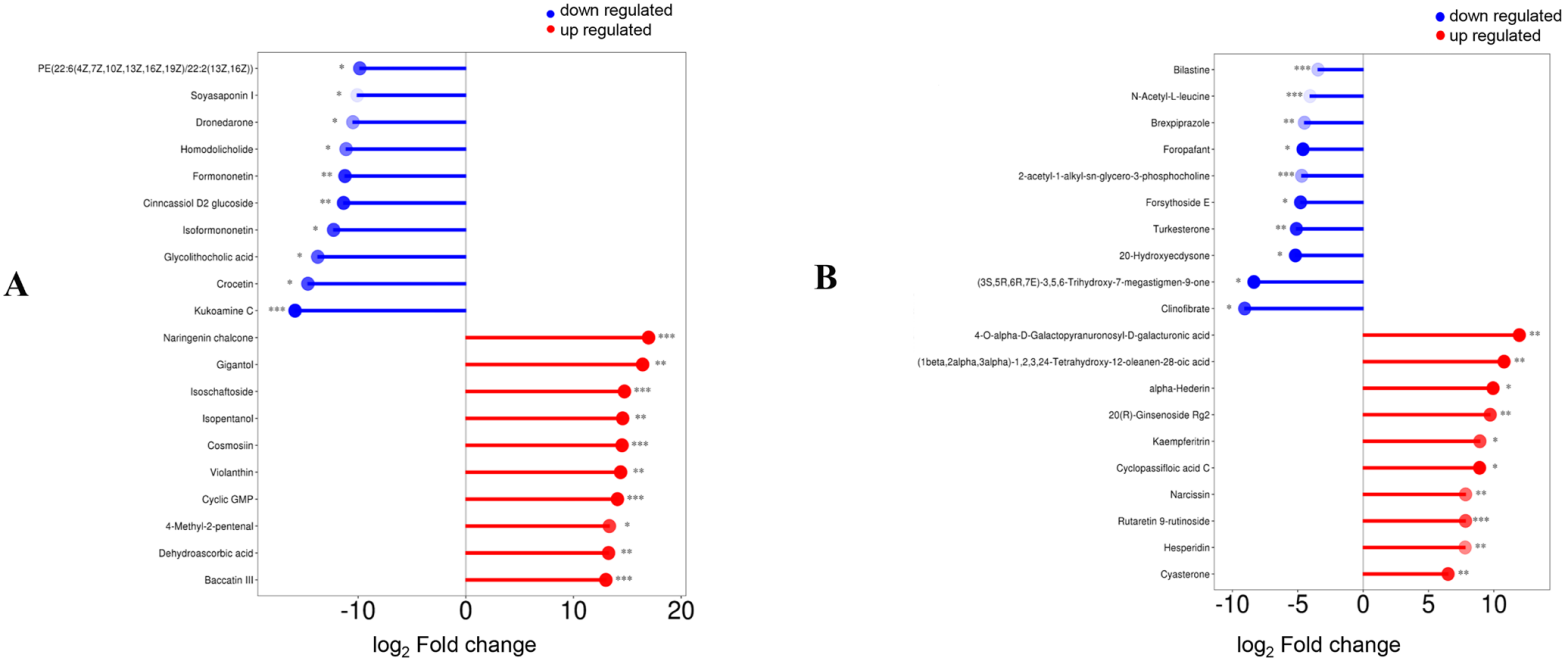
Distinctive nonvolatile microbial metabolites as well as the top 10 upregulated/downregulated metabolites produced by GMH (A) and GMD (B) after fermentation with DOP. ***p < 0.001, **p < 0.01, *p < 0.05, compared with the corresponding blank media group.

A growing number of studies have found that polysaccharides isolated from natural food could not only regulate gut microbiota in the host but also interplay with them to generate various microbial metabolites. Alteration of gut microbiota as well as their metabolites was demonstrated to be associated with a range of physiological processes in human body (Yang, Chen, et al. 2020; Yang, Yang, et al. 2020). In our current study, the effect of DOP on nonvolatile microbial metabolites generated by GMH and GMD was first evaluated by untargeted metabolomic analysis to provide insights into the regulatory effect of DOP on the intestinal microenvironment of host under healthy and diabetic status. As was shown in Figure 2A, in comparison with those from Blank media‐GMH group, about 20,521 significantly distinctive metabolites were identified in the DOP‐GMH group after 48 h fermentation, among which 13,635 metabolites were upregulated and 6886 metabolites were downregulated. Similarly, about 10,766 distinctive metabolites (Figure 2B) were identified in the DOP‐GMD group compared with those from the Blank media‐GMD group, among which 6707 metabolites were upregulated and 4059 metabolites were downregulated.

Furthermore, these identified nonvolatile metabolites were annotated based on the KEGG database to reveal their potentially relevant physiological pathways. As shown in Table 2, these distinctive metabolites identified in the DOP‐GMH group could be attributed to 12 KEGG pathways including two upregulated pathways like tryptophan metabolism pathway and 10 downregulated pathways such as D‐amino acid metabolism pathway. Previous studies have demonstrated that the upregulated tryptophan metabolism pathway could lead to the protective effect on cognitive function (Parolisi et al. 2023), while the downregulated D‐amino acid metabolism pathway could improve the immune system of the host (Liu, Wu, and Wang 2019). Therefore, the regulatory effect of DOP on GMH as well as their metabolites suggested that DOP could serve as the prebiotic to improve host immunity and cognitive function to further maintain the physiological status of healthy host.

**TABLE 2:** KEGG pathway annotated based on the significantly distinctive metabolites produced by GMH and GMD after fermentation with DOP.

Attributed pathways	GMH		GMD	
	Identified differential metabolite number	Differential abundance score (DA score)	Identified differential metabolite number	Differential abundance score (DA score)
Metabolic pathways	100	−0.06	90	+0.68
Biosynthesis of secondary metabolites	51	−0.68	42	+0.54
ABC transporters	19	−0.22	13	+0.56
Biosynthesis of amino acids	14	−0.59	14	+0.70
D‐amino acid metabolism	12	−0.71	12	+0.51
Purine metabolism	9	−0.13	8	+0.78
Primary bile acid biosynthesis	8	+0.03	7	+0.98
Carbon metabolism	7	−0.45	3	+0.73
Glucagon signaling pathway	6	−0.38	4	+0.95
Pyruvate metabolism	5	−0.23	4	+0.75
Tryptophan metabolism	4	+0.13	4	+0.75
Propanoate metabolism	5	−0.09	7	+1.13

Besides KEGG analysis on the metabolites in the DOP‐GMH group, the distinctive metabolites in the DOP‐GMD group were also analyzed. Twelve pathways were annotated, with all of them being upregulated, including linoleic acid metabolism, glucagon signaling pathway, and 2‐oxocarboxylic acid metabolism. Previous findings reported that the upregulation of linoleic acid metabolism might result in the alleviation of metabolic syndrome (Wen, Shang, and Wang 2022), and the pathway of 2‐oxocarboxylic acid metabolism was involved in the functional activation of pancreatic islets to improve glucose homeostasis (Wang, Jiang, et al. 2023; Wang, Wang, et al. 2023; Wang, Zhang, et al. 2023). In addition, Filippi et al. (2013) found that the glucagon signaling pathway could trigger the brain signal to regulate energy and glucose homeostasis. Our current metabolomic analysis indicated that the presence of DOP could upregulate linoleic acid metabolism pathway, glucagon signaling pathway, and 2‐oxocarboxylic acid metabolism pathway in the fermentation with GMD, suggesting that DOP might exhibit beneficial effects on the diabetic host through regulating gut microbiota as well as their metabolites.

Based on the above KEGG results from DOP‐GMH and DOP‐GMD groups, it was found that DOP not only was involved in the pathways of improving immunity and cognitive function of healthy host but also regulated the pathways of glucose homeostasis and functional activation of pancreas in diabetic host, providing evidence for the broad application of DOP as prebiotics to be consumed by both healthy and diabetic individuals.

#### 3.2.2 Different Regulatory Effects of DOP on SCFA Produced by GMH and GMD

**FIGURE 3:**
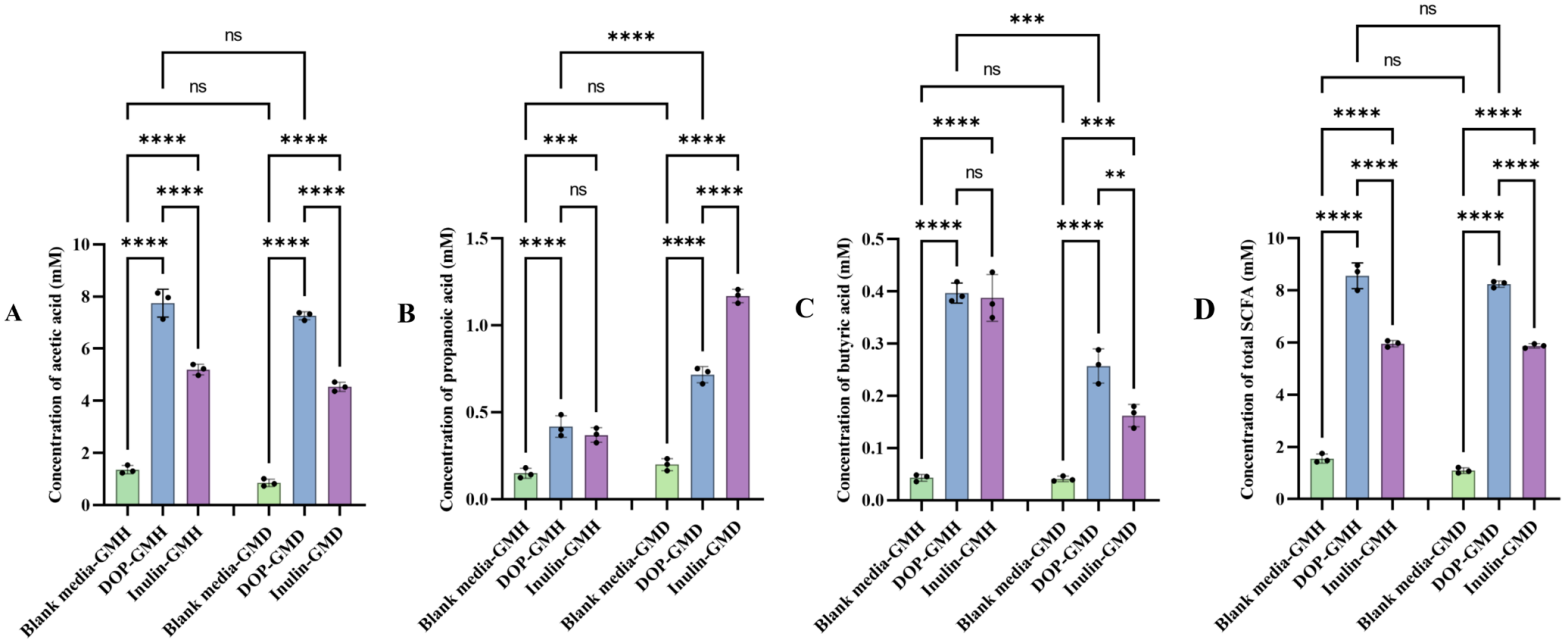
Comparison on the levels of acetic acid (A), propanoic acid (B), butyric acid (C), and total short‐chain fatty acids (D) produced by GMH and GMD after fermentation with DOP. ****:*p* < 0.0001, ***:*p* < 0.001, **:*p* < 0.01, *:*p* < 0.05, ns: no significant difference.

SCFA are a group of microbial volatile metabolites, which were reported to be associated with various biological activities such as anti‐inflammation and hypoglycemic effect (Xue et al. 2024; Yang, Fu, et al. 2024; Yang, Li, et al. 2024). In this study, the concentration of three main SCFA (acetic acid, propanoic acid, and butyric acid) in the fermentation broth were quantified by GC–MS, and the results shown in Figure 3 indicated that levels of these SCFA were all increased significantly in the DOP broth after fermentation with GMH or GMD compared with those of blank media. In comparison with inulin, a common prebiotic used to improve glucose homeostasis and intestinal microenvironment, DOP could lead to significantly higher levels of acetic acid and total SCFA after fermentation with GMH or GMD, suggesting that DOP might be another impressive prebiotic used to improve intestinal microenvironment.

Although total SCFA levels in blank media or DOP broth fermented with GMH were not significantly different from that with GMD, the propanoic acid level in DOP broth fermented with GMD was significantly higher while its butyric acid level was significantly lower than those with GMH. Based on previous reported differential biological activities of the three SCFA such as decrease of total cholesterol and triglyceride levels by acetate and propionate (Hu, Nie, and Xie 2018) and improvement of intestinal integrity to prevent influx of detrimental metabolites by sodium butyrate (Hung and Suzuki 2018; Song, Cheng, et al. 2024; Song, Zou, et al. 2024), our observed differentially regulatory effect of DOP on three individual SCFA produced by GMH and GMD implied that the biological activity of DOP might depend on the pathological status of the host.

### 3.3 Different Regulatory Effects of DOP on Compositions of GMH and GMD

**FIGURE 4:**
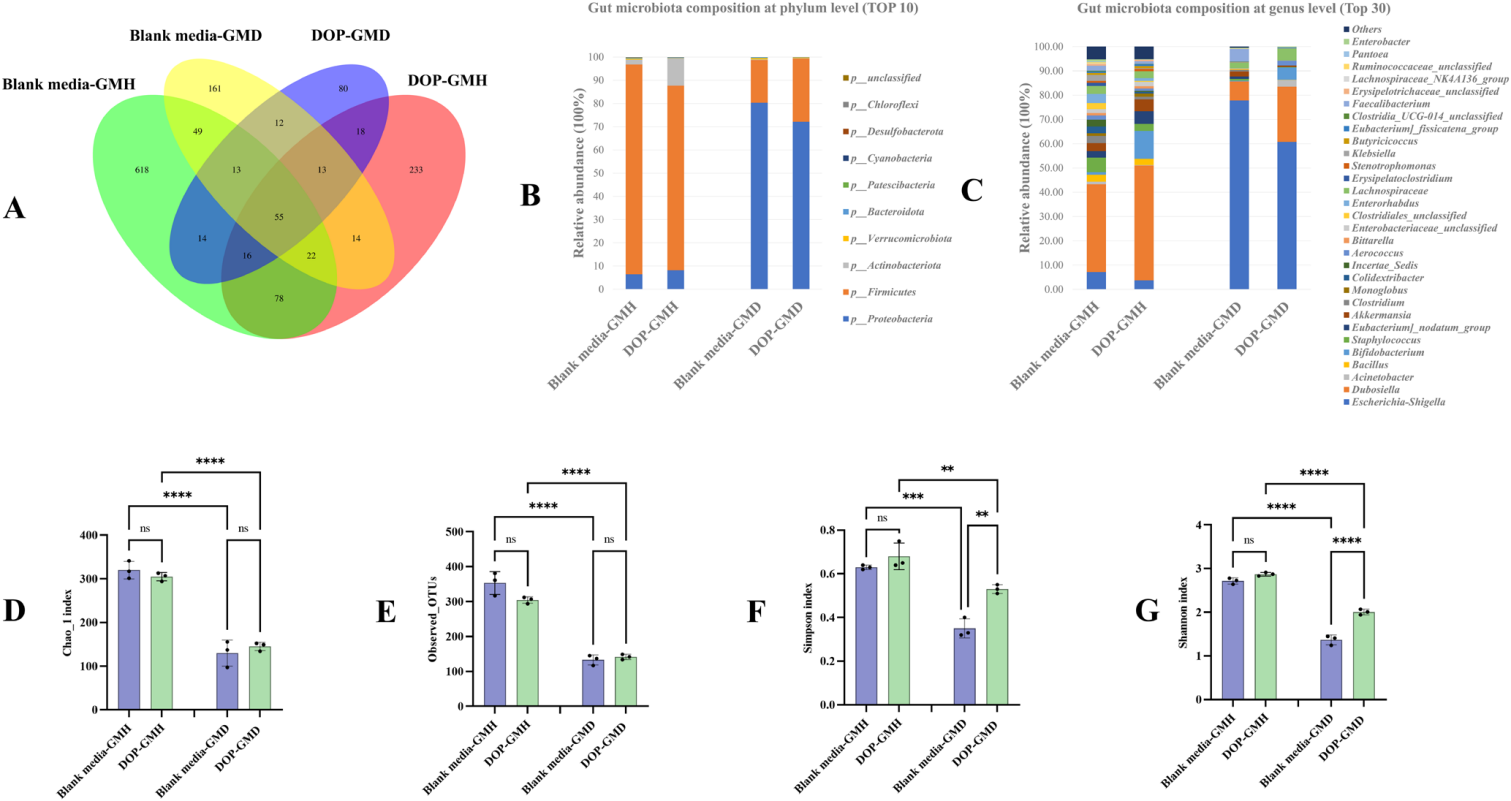
Effect of DOP on the composition of GMH and GMD as demonstrated in Venn Diagrams of overlapping and exclusive ASVs clustering (A), relative abundance at the phylum level (B) and genus level (C), and biodiversity analysis (D–G). ****p < 0.0001, *** *p* < 0.001, **p < 0.01, ns: no significant difference.

Recent studies found that most edible polysaccharides exhibited numerous beneficial effects by regulating gut microbiota (Zhang et al. 2021). Since DOP was commonly consumed as a functional food by both healthy and diabetic individuals, the effect of DOP on the composition of GMH and GMD was evaluated and compared herein. Gut microbiota composition analysis at the phylum level shown in Figure 4B revealed that treatment of DOP promoted the growth of *Actinobacteriota* and *Firmicutes* in GMH and GMD, respectively, suggesting a differentially regulatory potential of DOP on the composition of GMH and GMD at the phylum level. Relative abundance analysis of gut microbiota at the genus level shown in Figure 4C indicated that the bacteria composition was different among the four treatment groups (Blank media‐GMH, DOP‐GMH, Blank media‐GMD, and DOP‐GMD) with specific distinctive intestinal bacteria shown in Figure S6 by LEfSe analysis.

Biodiversity analysis shown in Figure 4D–G found that the Observed_OTUs, Chao_1 index, Shannon index, and Simpson index in GMH were significantly higher than those in GMD after 48 h fermentation with blank media, which was supported by previous findings on the decreased biodiversity in gut microbiota from the diabetic host (Wang et al. 2022). Furthermore, in comparison with blank media treatment, either the richness (indicated by Observed_OTUs and Chao_1 index) or the diversity (indicated by Shannon index and Simpson index) in GMH showed insignificant changes after DOP treatment, implying that DOP was unable to improve the biodiversity of GMH. However, the significant improvement on diversity of GMD was observed after DOP treatment although its richness was not improved significantly, suggesting that DOP exhibited potential to improve the impaired gut microbial diversity of the diabetic host.

**FIGURE 5:**
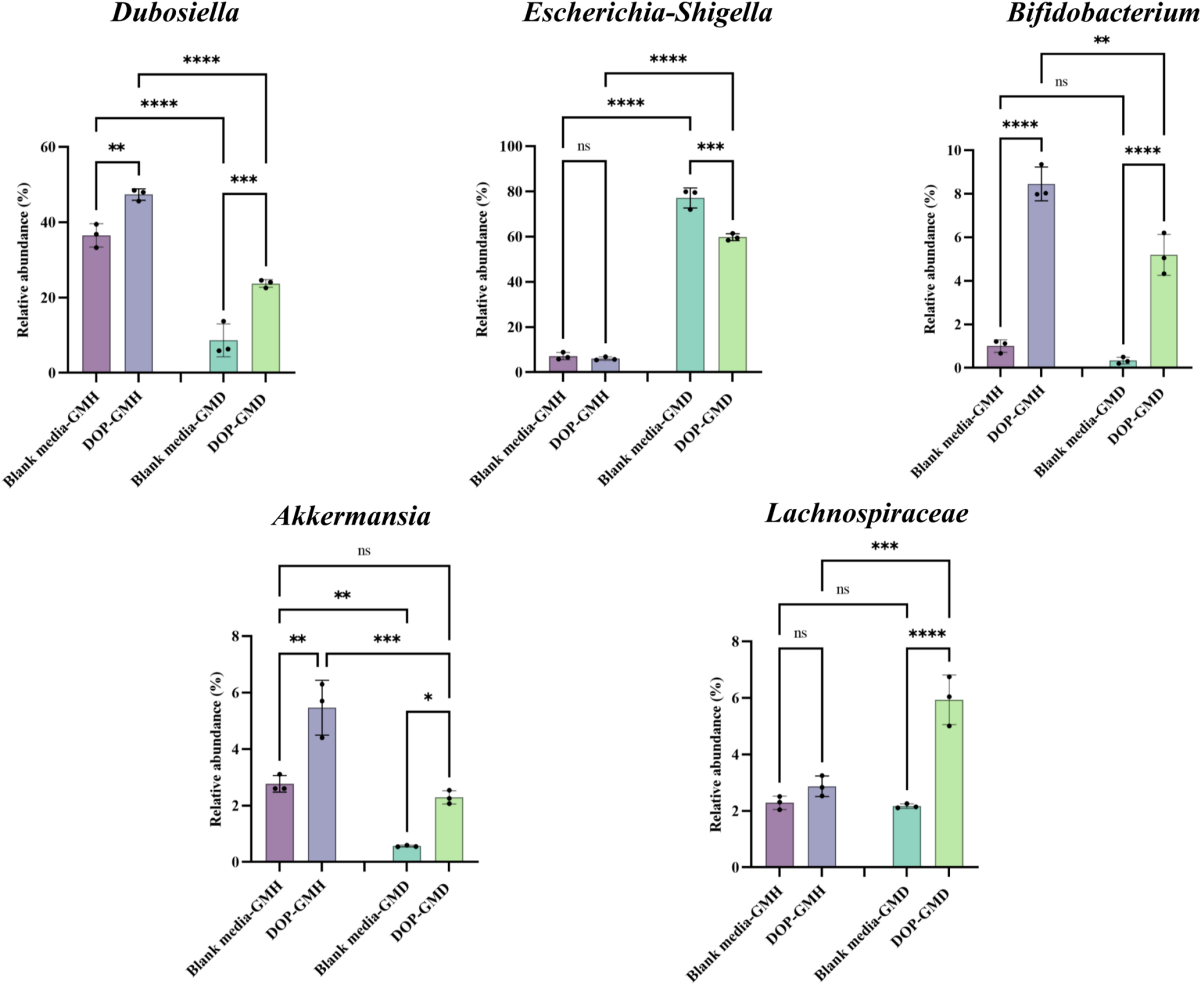
Effect of DOP on the relative abundance of top 5 intestinal microflora (*Dubosiella*, *Escherichia‐Shigella*, *Bifidobacterium*, *Akkermansia*, and *Lachnospiraceae*) under healthy and diabetic status. **p < 0.01, ***p < 0.001, ****p < 0.0001, ns, no significant difference.

Further analysis on the intestinal flora with top five abundances (Figure 5) demonstrated that the abundance of *Dubosiella* and *Akkermansia* was significantly lower, while the abundance of *Escherichia‐Shigella* was significantly higher in GMD than those in GMH in the fermentation of blank media, which coincided with previous clinical findings on more abundant *Escherichia‐Shigella* in T2D individuals (Baltazar‐Díaz et al. 2022). After treatment with DOP, the growth of *Dubosiella*, *Akkermansia*, *Lachnospiraceae*, and *Bifidobacterium* in GMD was promoted significantly, while the growth of *Escherichia‐Shigella* was inhibited significantly in comparison to those treated with blank media, suggesting that treatment with DOP not only inhibited the growth of pathogens but also benefited the growth of probiotics in GMD. Similarly, DOP treatment could significantly promote the growth of probiotics (*Dubosiella*, *Akkermansia*, and *Bifidobacterium*) in GMH, while the significantly inhibitory effect on pathogens (*Escherichia‐Shigella*) was not observed. Such a different regulation by DOP on compositions in GMH from that in GMD implied that DOP might possess the potential to make probiotics robust in both healthy and diabetic hosts while inhibit pathogen only in the diabetic host. Notably, the significant difference on the abundance of *Akkermansia* between GMH and GMD was diminished after treatment with DOP, implying that DOP could even reverse the insufficient abundance of *Akkermansia* in GMD back to normal. Despite the abovementioned health‐promoting potential of DOP on gut microbiota, it should be noted that gut microbiota composition in mice differed from that in human (Fredric et al. 2005); thus, our current observations on gut microbiota from mice warrant further verification in fecal samples from human.

### 3.4 Correlation Analysis Between Gut Microbiota Under Different Status and Their Metabolites After Fermentation With DOP

**FIGURE 6:**
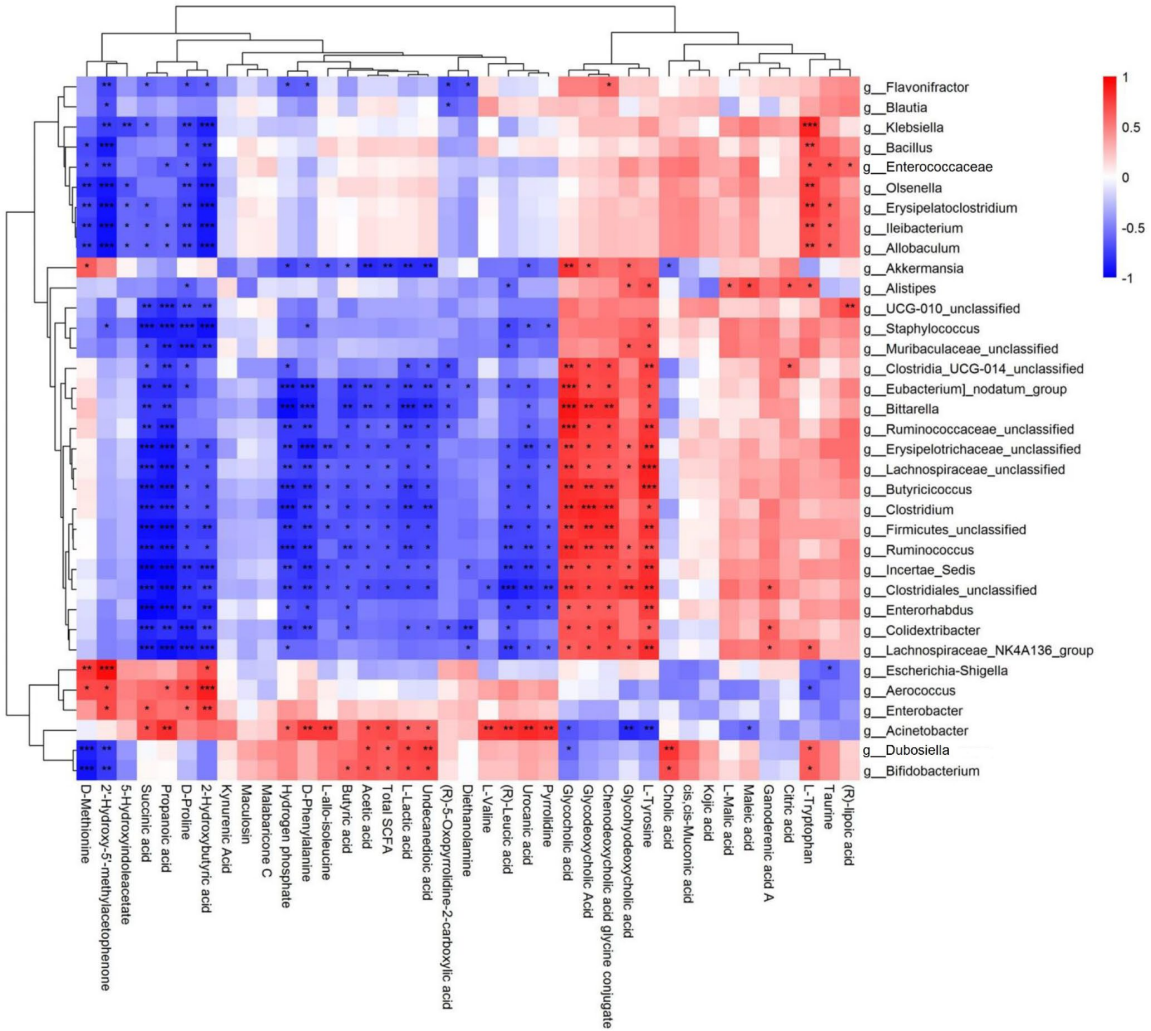
Correlation analysis between microbial metabolites and gut microbiota in different treatment groups (Blank media‐GMH, DOP‐GMH, Blank media‐GMD, and DOP‐GMD). ***p < 0.001, **p < 0.01, *p < 0.05, ns, no significant difference.

Microbial metabolites played a crucial role in various physiological processes in human body and might participate in the communication between gut microbiota and their host (Hou et al. 2022). Gut microbiota analysis in the present study has shown different regulatory effects of DOP on GMH and GMD. To further investigate the relationship between altered gut microbiota and microbial metabolites, an integrative correlation analysis (Figure 6) was conducted between the abundance of gut microbiota (Top 35) and levels of nonvolatile metabolites (Top 35) and SCFA among the four treatment groups (Blank media‐GMH, DOP‐GMH, Blank media‐GMD, and DOP‐GMD).

On one hand, the abundance of *Escherichia‐Shigella*, one dominant intestinal pathogen in diabetic host, was positively correlated with levels of 2′‐hydroxy‐5′‐methylacetophenone, D‐methionine, and 2‐hydroxybutyric acid (*p* < 0.05) while negatively correlated with that of taurine (*p* < 0.05). Previous study has found that D‐methionine could be transformed into taurine by intestinal microorganisms (Franconi et al. 2006; Wang et al. 2024). Since our correlation analysis showed that the abundance of *Escherichia‐Shigella* was positively correlated with D‐methionine level and negatively correlated with taurine level, it was speculated that *Escherichia‐Shigella* might lack the potential to transform D‐methionine into taurine. Taurine was demonstrated to be able to regulate disordered lipid metabolism and improve insulin sensitivity, whereas excessive accumulation of D‐methionine might deteriorate insulin sensitivity and lipid metabolism function of the host (Yin et al. 2018; Wang, Jiang, et al. 2023; Wang, Wang, et al. 2023; Wang, Zhang, et al. 2023). Gut microbiota composition analyses in the current in vitro fermentation study also found that DOP treatment could decrease the abundance of *Escherichia‐Shigella* in GMD, which was consistent with our previous studies on *db*/*db* mice (Song, Cheng, et al. 2024; Song, Zou, et al. 2024; Zou et al. 2024). Consequently, it was proposed that DOP treatment might inhibit the growth of *Escherichia‐Shigella* to indirectly restore the transformation of D‐methionine to taurine to subsequently exhibit its beneficial effect on cognitive function and glucose metabolism of diabetic host. Furthermore, 2‐hydroxybutyric acid level was revealed to be positively correlated with the abundance of *Escherichia‐Shigella* (*p* < 0.05), which was consistent with previous clinical findings on higher 2‐hydroxybutyric acid level and *Escherichia‐Shigella* abundance in T2D (Sousa et al. 2021).

On the other hand, Spearman's correlation analysis showed that the abundance of *Dubosiella* and *Bifidobacterium*, two major probiotics in healthy host (Arboleya et al. 2016; Gavzy et al. 2023), was positively correlated with levels of L‐tryptophan, undecanedioic acid, L‐lactic acid, and cholic acid (*p* < 0.05) while negatively correlated with that of D‐methionine, 2′‐hydroxy‐5′‐methylacetophenone, and glycocholic acid (*p* < 0.05). Since L‐lactic acid was previously demonstrated to improve the immunosuppression of host by activating GPCR81 receptors (Luo et al. 2022) and L‐tryptophan, as an important aromatic amino acid, it was found to exhibit the beneficial effect on lipid metabolism and cognitive function (Pan et al. 2024). The current findings on the increased levels of lactic acid and tryptophan after DOP fermentation with GMH might support a series of in vivo studies reporting the beneficial potential of DOP as functional food on the health of host (Sun et al. 2022). Moreover, the abundance of *Dubosiella* was positively correlated with acetic acid level and total SCFA level while the abundance of *Bifidobacterium* was positively correlated with acetic acid level, butyric acid level, and total SCFA level. These correlation analysis results were supported by our previous in vivo study that the relative abundance of *Bifidobacterium* was positively correlated with the total SCFA level in *db*/*db* mice (Lyu et al. 2021).

**FIGURE 7:**
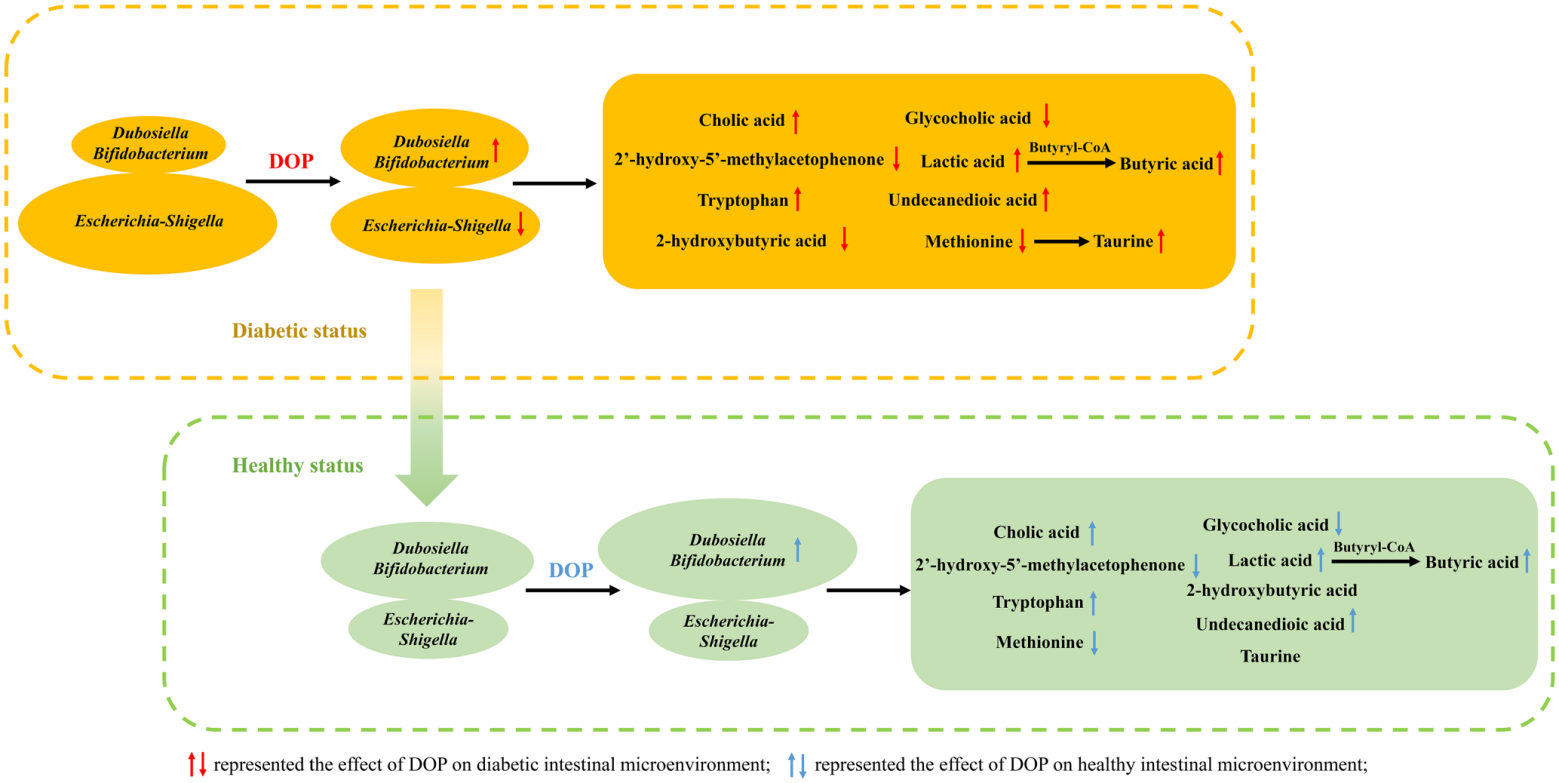
Proposed regulatory pathway of DOP on gut microbiota as well as microbial metabolites under healthy (green) and diabetic (yellow) status.

Based on the above findings, the potential regulatory pathway of DOP on gut microbiota and its metabolites is proposed in Figure 7. Under diabetic status, DOP could inhibit the *Escherichia‐Shigella* growth and promote probiotics’ (such as *Dubosiella* and *Bifidobacterium*) growth in the diabetic host, leading to generating more bioactive microbial metabolites including butyric acid and taurine to exhibit the beneficial effect on glucose homeostasis. Under healthy status, it was proposed that DOP could promote probiotics’ growth resulting in more production of tryptophan and lactic acid to further exhibit the beneficial effect on the immune system and cognitive function. Collectively, our current findings provided new insights into the health‐promoting potential of DOP on the host under different status based on its interplay with gut microbiota.

## 4 Conclusions

The present study for the first time revealed that the metabolic signatures and regulatory effect of DOP on gut microbiota depended on the pathological status of the host. Mannose in DOP was more utilized by GMD with a higher production of propanoic acid and lower production of butyric acid in comparison with those by GMH. It is noteworthy that DOP could promote probiotic growth under both healthy and diabetic status while only inhibited pathogens under diabetic status.

## Author Contributions

**Qianbo Song:** data curation (lead), formal analysis (lead), writing – original draft (lead). **Junju Zou:** data curation (equal), formal analysis (lead), methodology (lead). **Sau Wan Cheng:** formal analysis (equal), methodology (equal). **Kendra Sek Lam Li:** formal analysis (equal), methodology (equal). **David Tai Wai Lau:** formal analysis (supporting). **Xiao Yang:** resources (equal). **Pang Chui Shaw:** funding acquisition (equal), resources (equal). **Zhong Zuo:** conceptualization (lead), funding acquisition (lead), project administration (lead), supervision (lead), writing – review and editing (lead).

## Conflicts of Interest

The authors declare no conflicts of interest.

## Supporting information


Data S1.

## Data Availability

The data used and/or analyzed during this study are available from the corresponding author upon reasonable request.
