# Supplementary material for: Insights Into Metabolic Signatures and Regulatory Effect of Dendrobium officinale Polysaccharides in Gut Microbiota: A Comparative Study of Healthy and Diabetic Status
Source: Food Sci Nutr. 2024 Dec 6;13(1):e4651. doi: 10.1002/fsn3.4651 (PMC11717035; doi:10.1002/fsn3.4651)
Supplement: Supplementary file 1 — Data S1. [file FSN3-13-e4651-s001.pdf]

Supplementary Material

Insights into metabolic signatures and regulatory effect of *Dendrobium officinale* polysaccharides in gut microbiota: A comparative study of healthy and diabetic status

Qianbo Song[1,#], Junju Zou[1,2,4,5,#], Sau Wan Cheng[2,4], Kendra Sek Lam Li[3], David Tai Wai Lau[4], Xiao Yang[3], Pang Chui Shaw[2,4], Zhong Zuo[1,2] *

[1]*Guangdong-HongKong-Macao Joint Laboratory for New Drug Screening, School of Pharmacy, The Chinese University of Hong Kong, Shatin, Hong Kong SAR, P. R. China.*

[2]*State Key Laboratory of Research on Bioactivities and Clinical Applications of Medicinal Plants, The Chinese University of Hong Kong, Shatin, Hong Kong SAR, P. R. China.*

[3]*Department of Microbiology, The Chinese University of Hong Kong, Shatin, Hong Kong SAR, P. R. China.*

[4]*School of Life Sciences and Li Dak Sum & Yip Yio Chin Research and Development Centre for Chinese Medicine, The Chinese University of Hong Kong, Shatin, Hong Kong SAR, P. R. China.*

[5]*School of Traditional Chinese Medicine, Hunan University of Chinese Medicine, Chang Sha, P. R. China.*

*Corresponding author:
Dr. Zhong Zuo
School of Pharmacy, Faculty of Medicine,
The Chinese University of Hong Kong, Shatin, N.T., Hong Kong SAR.
E-mail: joanzuo@cuhk.edu.hk
Phone: +852 3943 6862
Fax numbers: +852 2603 5295

#: The authors contributed equally to the work.

Methodology

Fourier Transform Infrared (FT-IR) analysis of *Dendrobium officinale* polysaccharides (DOP)

DOP (2 mg) was mixed uniformly with 150 mg spectroscopic grade potassium bromide (KBr) powder in a dry environment. The mixture was powdered in a blender and then pressed into a pellet. The FT-IR spectrum was detected in a Bruker Fourier transform infrared spectrometer with the wavenumber of 4000-400 cm^{-1}.

In-vitro digestion of DOP in the simulated gastrointestinal fluid

The in-vitro simulated digestion experiment was divided into gastric digestion and small intestinal digestion. Firstly, 20 mL DOP aqueous solution (6 mg/mL) was mixed with simulated gastric fluid (*v:v*, 1:1) and kept at thermostatic shaker (37 ℃, 100 rpm) for 2 h incubation. The mixture of deionized water and simulated gastric solution was used as blank control. After incubation, the obtained gastric digested DOP solution was adjusted to pH 7.0 by sodium hydroxide and then mixed with simulated small intestinal solution (*v:v*, 10:3) for another 6 h incubation at thermostatic shaker (37 ℃, 100 rpm). The mixture of deionized water and simulated small intestinal solution was used as blank control. After digestion, all samples were inactivated enzyme activity immediately by immersing into boiling water for 10 min. The digestion was performed in triplicate and all the sample were collected and stored at -80 ℃ for further analysis.

Reducing sugar content measurement

Standard glucose/sample solution was mixed with 3,5-dinitrosalicylic acid (DNS) solution (*v:v*, 1:1). Standard glucose solution (5 mg/mL) was serially diluted into different concentrations to plot standard curve. The reducing sugar content was calculated as follows:

$$\text{Reducing sugar content (mg/mL)} = \frac{\text{The reducing sugar content in sample solution (mg)} - \text{The reducing sugar content in blank solution (mg)}}{\text{The sample volume (mL)}}$$

Table S1. Recipe of general anaerobic media

Ingredient	Amount
Peptone	2.0 g
Yeast extract	2.0 g
NaCl	0.1 g
KH_2PO_4	0.04 g
K_2HPO_4	0.04 g
$MgSO_4$	0.01 g
$CaCl_2$	0.01 g
$NaHCO_3$	2.0 g
Cysteine-HCl	0.5 g
Bile salt	0.5 g
Tween 80	2 mL
Water	1 L

Table S2. Recipe of simulated gastric fluid and small intestinal fluid

**Simulated gastric fluid**		**Simulated small intestinal fluid**	
Ingredient	Amount	Ingredient	Amount
NaCl	465 mg	NaCl	135 mg
$CaCl_2$	22.5 mg	$CaCl_2$	8.5 mg
KCl	165 mg	KCl	16.5 mg
$NaHCO_3$	90 mg	pancreatin (4000 U/g)	700 mg
gastric lipase (100000 U/g)	37.5 mg	bile salt	800 mg
pepsin (3000 U/mg)	35.4 mg	trypsin (2500 U/mg)	2.6 mg
water	150 mL (pH 2.0)	water	80 mL (pH 7.5)

Table S3. Mobile phase gradient program for untargeted metabolomics analysis by UPLC-Orbitrap Mass Spectrometer

Time (min)	Acetate ammonium buffer (A)	Acetonitrile (B)
0-0.7	99%-99%	1%-1%
0.7-9.5	99%-1%	1%-99%
9.5–12.0	1%-1%	99%-99%

Table S4. GC-MS chromatographic condition for short chain fatty acids (SCFA) analysis

SCFA analysis was conducted on a Shimadzu QP2010 GC/MS system coupled with an Agilent J & W fused silica capillary column (DB-FFAP, 30 m, 0.32 mm ID, 0.25 μm film thickness) and the analytical parameters were set as follows:

Item	Parameters
Column temperature program	70 ℃, kept for 1 min<br>70 ℃ to 230 ℃, rate 5 ℃ / min<br>230 ℃, kept for 5 min
Injector temperature	230 ℃
Transfer line temperature	230 ℃
Ion source temperature	200 ℃
Sample injection volume	1 μL
Carrier gas	Helium (99.999%)
Linear velocity on column	36.7 cm/sec
Mass mode	Quadrupole mass spectrometer
Ionization mode	Electron impact (EI)
Electron energy	-70 eV
Ion polarity	Positive
Mass range (m/z)	50-650
Scan time	0.5 sec
Electron multiplier voltage	1.1 kV

Results

Table S5. Chemical characterization of DOP

Origin	Total sugar content of the sample	Molar ratio of mannose/glucose
Huoshan, Anhui, China	90.8±2.7%	2.12

Table S6. Number of the distinctive non-volatile microbial metabolites produced by GMH and GMD after fermentation with DOP

Type	Number of metabolites without significant alteration	Number of up-regulated metabolites	Number of down-regulated metabolites
**GMH**	30066	13635	6886
**GMD**	39821	6707	4059

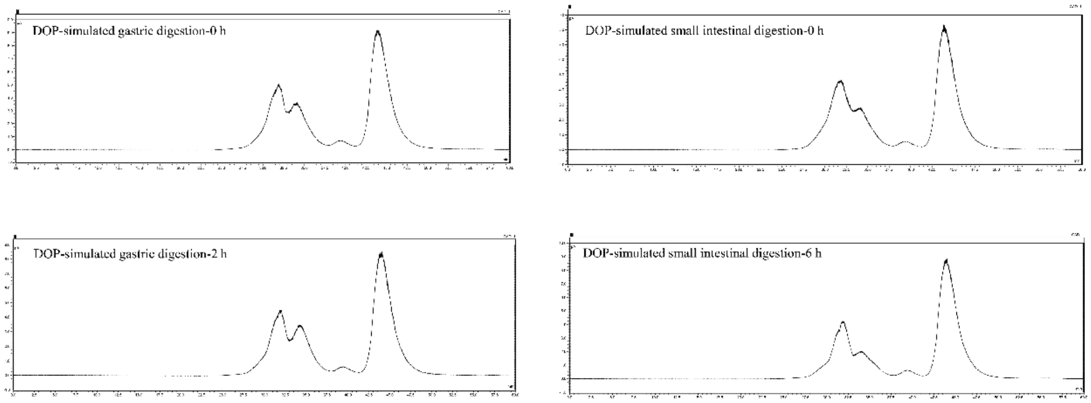

Reducing sugar level alteration of DOP after gastrointestinal digestion

	Timepoints	Concentration of reducing sugar (mg/mL)
Simulated gastric fluid digestion	0 h	0.459±0.011
	2 h	0.504±0.013
Simulated small intestinal fluid digestion	0 h	0.416±0.009
	2 h	0.433±0.013
	4 h	0.445±0.018
	6 h	0.473±0.010

Figure S1. Molecular weight distribution and reducing sugar level of DOP broth before and after gastrointestinal digestion

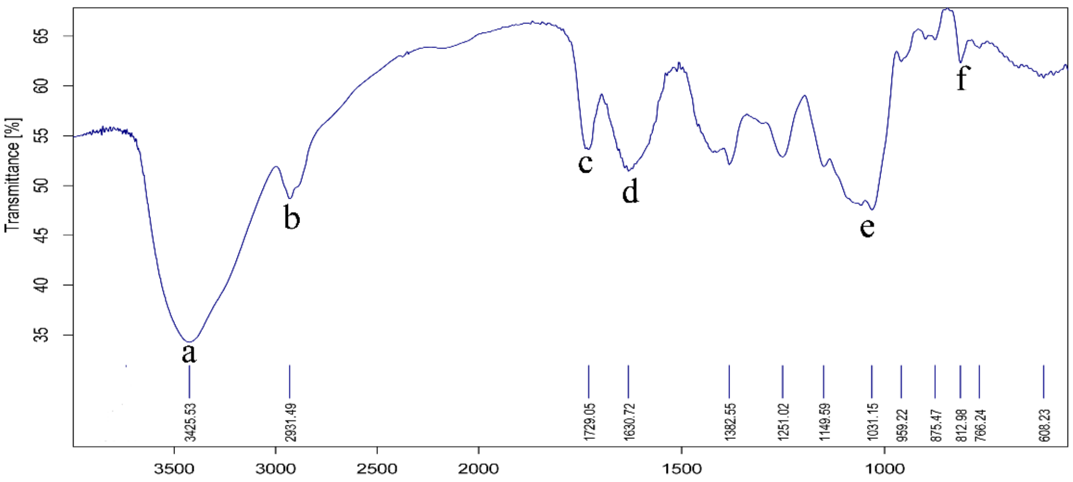

Number	Wavenumber(cm^{-1})	Possible functional group
a	3430-3420	O-H
b	2940-2910	C-H
c	1735-1725	C=O stretching
d	1660-1610	absorbed water
e	1200-1000	C-OH, C-O-C
f	900-810	β-type glycosidic linkages

Figure S2. FT-IR spectrum of DOP

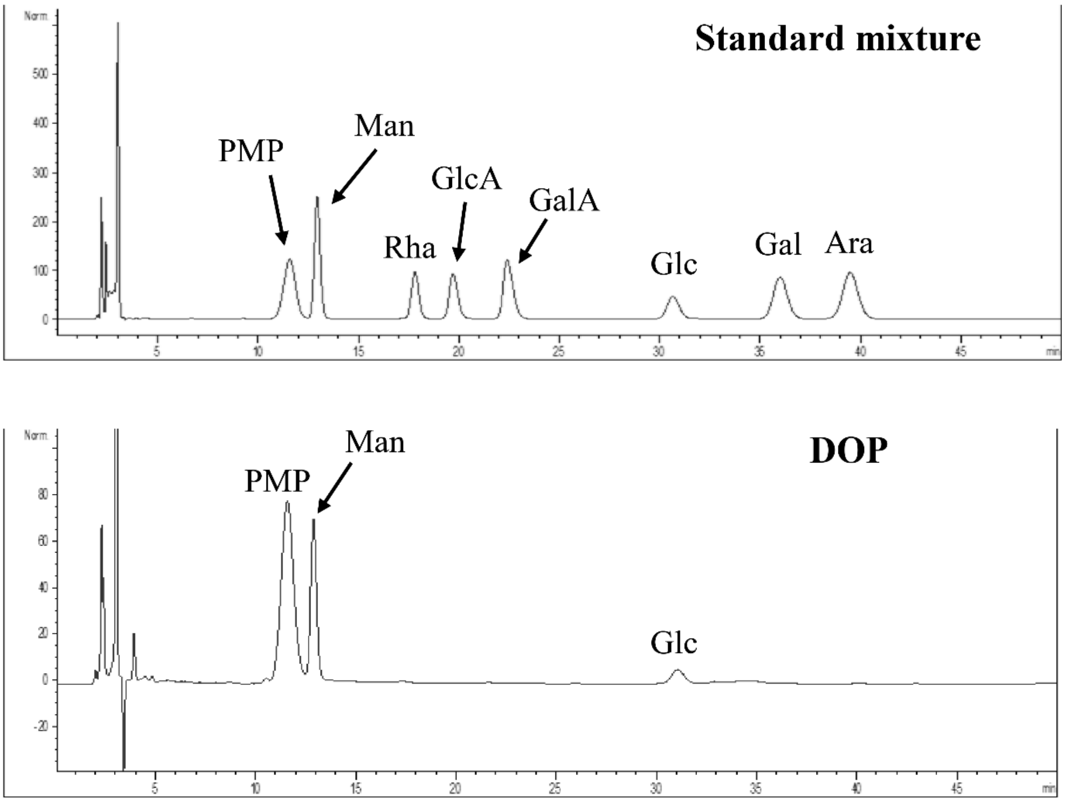

Figure S3. Chromatograms for monosaccharides composition of DOP

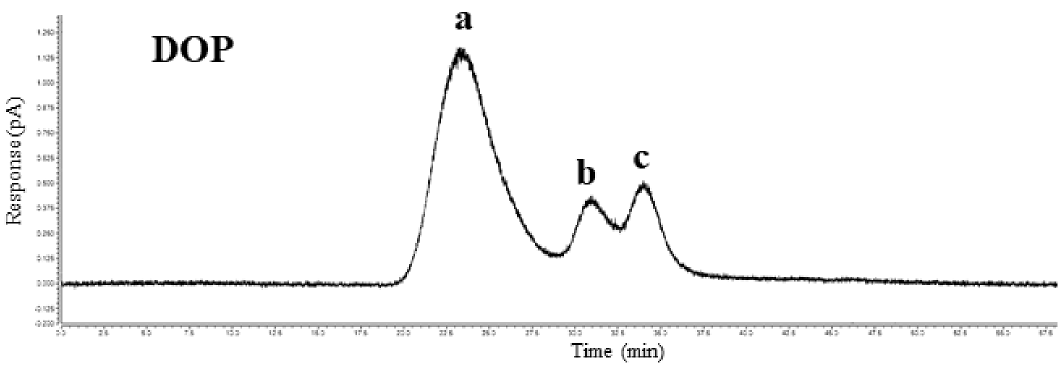

Peak No.	Retention time (min)	Molecular weight (Da)
a	~23.3	$\sim 6\times10^5$
b	~31.5	~1600
c	~34.0	~300

Figure S4. Chromatograms for molecular weight distribution of DOP

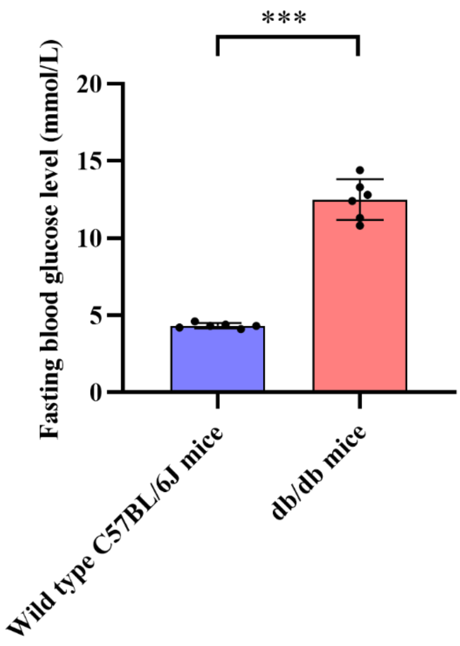

Figure S5. Fasting blood glucose level of wild type C57BL/6J mice and *db/db* mice at the age of 6-week without any interference.

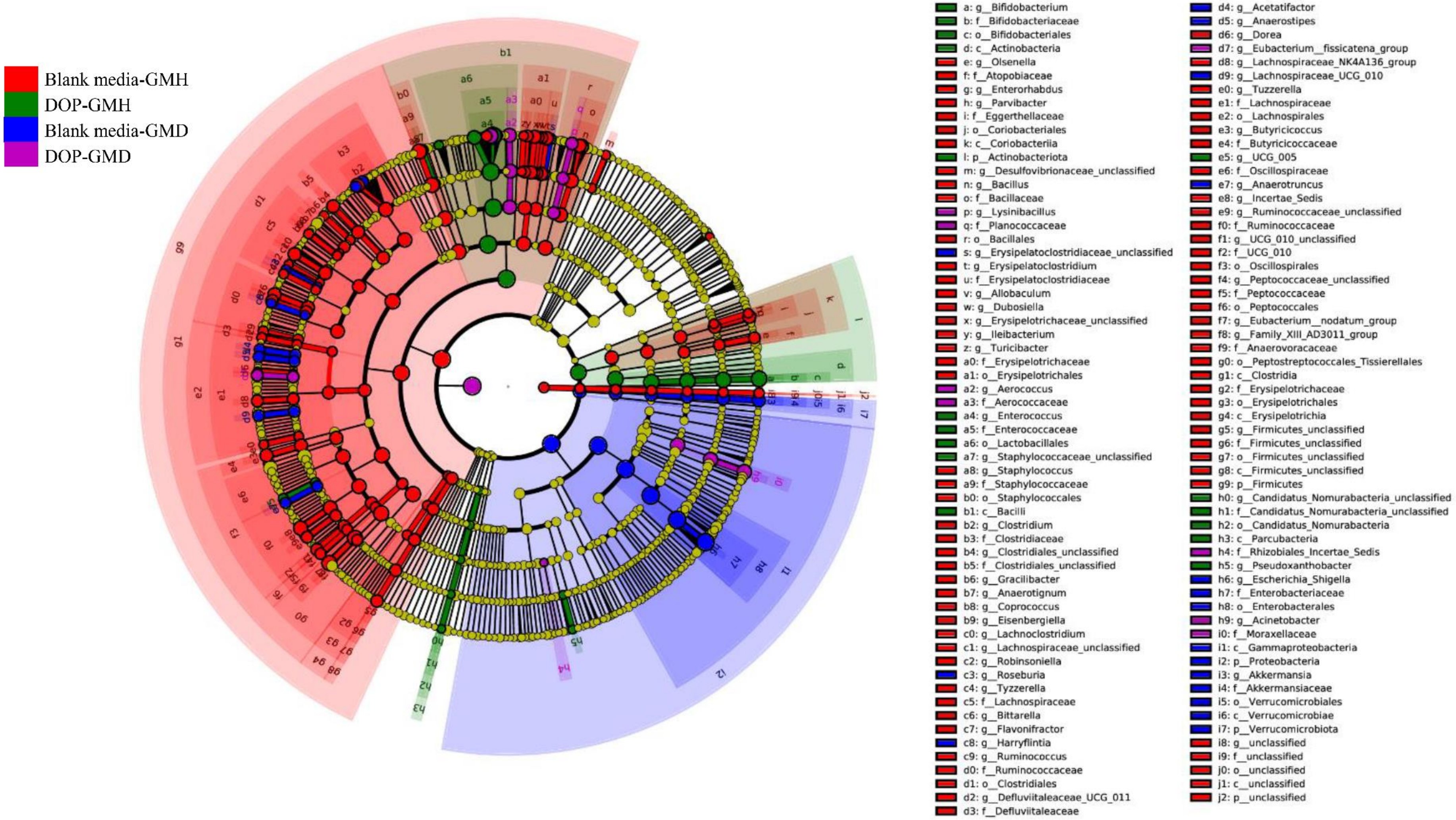

Figure S6. Effect of DOP on composition of GMH and GMD as demonstrated by LEfSe analysis
